# Supplementary material for: New suspension-feeding radiodont suggests evolution of microplanktivory in Cambrian macronekton
Source: Nat Commun. 2018 Sep 14;9:3774. doi: 10.1038/s41467-018-06229-7 (PMC6138677; doi:10.1038/s41467-018-06229-7)
Supplement: Supplementary file 1 — Supplementary Information [file 41467_2018_6229_MOESM1_ESM.pdf]

**New suspension-feeding radiodont suggests evolution of microplanktivory in Cambrian macronekton**

Lerosey-Aubril et al.

# SUPPLEMENTARY INFORMATION

This file comprises:

**Supplementary Note 1.** Systematic Palaeontology

**Supplementary Note 2.** Phylogenetic analyses.

**Supplementary Fig. 1.** Exposures of the Drumian (Miaolingian) Wheeler Formation yielding *Pahvantia hastata*.

**Supplementary Fig. 2.** Cephalic carapace elements of *Pahvantia hastata*.

**Supplementary Fig. 3.** Taphonomic scenario explaining the observed morphology of the frontal appendage of *Pahvantia hastata*.

**Supplementary Fig. 4.** Variations of tree topology of parsimony analyses under implied weighting and various values of concavity constant k.

**Supplementary Fig. 5.** Results of the Bayesian inference analysis.

# SUPPLEMENTARY NOTE 1: SYSTEMATIC PALAEONTOLOGY

**Terminology.** The terms 'central element' and 'lateral element'[1] are used to describe the two parts of the cephalic carapace. These terms can be applied across hurdiids bearing a tripartite carapace, and are more general than those used specifically for *Hurdia* introduced by Daley et al.[2]. 'H-element' (central element) and 'P-element' (lateral element) refer to the fact that these sclerites were once regarded as carapaces of two distinct taxa ('H' for *Hurdia* and 'P' for *Proboscicaris*). Likewise, we followed Chen et al.[3] and Haug et al.[4] in using the anatomical term 'peduncle' to refer to the proximal region of the frontal appendages associated with no endite or a single reduced one. However, it is worth mentioning that we do not share these authors' view about the homologous relationship between radiodont frontal appendage and megacheiran great appendage – these organs are born by different cephalic segment (protocerebral and deutocerebral, respectively) and therefore not homologous. The term 'shaft'[5,6,7] was not preferred over 'peduncle', as it 1) has various meanings and 2) is generally applied to tools, not organs. Otherwise, the terminology essentially follows Cong et al.[8] for the morphology of the frontal appendage, and Briggs[9] for its orientation. Importantly, 'proximal(-ly)' and 'distal(-ly)' are used in reference to the larger and smaller extremities, respectively, of both the whole appendage and the endites. To avoid confusion between these two contexts, auxiliary spines occurring on the margin of an endite facing the proximal end of the appendage are described as 'posterior', the ones located on the margin facing the distal end of the appendage as 'anterior'[9]. Moreover, the height of a podomere refers to the distance between its dorsal and ventral margins, while its length corresponds to the distance between its boundaries with the preceding and following podomeres. Abbreviations: exs., exsagittally, sag., sagittally; tr., transverse.

**Order Radiodonta** Collins[10]

**Family Hurdiidae** fam. nov.

**Type genus.** *Hurdia* Walcott[11].

**Other genera included.** *Aegirocassis* Van Roy et al.[1], *Pahvantia* Robison & Richards[12], *Peytoia* Walcott[13], *Schinderhannes* Kühl et al.[14], *Stanleycaris* Pates et al.[15].

**Diagnosis.** Radiodonts with frontal appendages composed of proximal, intermediate, and distal regions: proximal region comprising one or more podomeres bearing small or no endites; intermediate region composed of five podomeres about twice higher than long, and bearing endites longer than podomere heights; distal region characterized by podomeres conspicuously reduced in height, length, or both. Endites plate-like, not alternating in size between odd and even podomeres, typically curved towards the appendage distal tip, and bearing auxiliary structures (spines or setae) projecting at a right angle from their anterior margins only. Oral cone comprises four large plates at right angles to each other.

**Occurrences.** Cambrian: Stage 3 Zawiszyn Formation (Poland[16]); Cambrian Stage 4 Balang (Hunan, China[17]) and possibly Shuijingtuo (Hubei, China[18]) formations; Wuliuan Spence Shale (Utah, USA; e.g.[19]), Burgess Shale and Stephen formations (British Columbia, Canada; e.g.[20]); Drumian Jince (Czech Republic[21]), Marjum and Wheeler (Utah, USA; e.g.[15,19], this study) formations; Furongian part of the Klonówka Shale Formation (Poland[22]). Ordovician: Tremadocian Lower Fezouata Formation (Morocco; e.g.[1]). Devonian: Emsian Kaub Formation (Germany[14]).

**Remarks.** A family Hurdiidae was first erected by Vinther et al.[23], but their definition of this taxon was not based on characters, but on purported phylogenetic relationships with a nominate taxon (i.e. *Hurdia victoria*; their 'phylogenetic taxonomy'). The International Code of Zoological Nomenclature (articles 13.1, 13.2) is clear on that matter: the description of a new taxon must be accompanied by a diagnosis, that is to say, 'a summary of the characters that differentiate the new nominal taxon from related or similar taxa' (see Recommendation 13A). In the absence of such a diagnosis, Vinther et al.'s family Hurdiidae[23] can only be regarded as a *nomen nudum*.

Since Vinther et al.'s pioneer study[23], a monophyletic clade Hurdiidae has been retrieved in all the cladistic analyses exploring the relationships within the Radiodonta[1,24,25,this study], despite significant changes to the original matrix of characters and the inclusion of new taxa (e.g. *Aegirocassis*, *Pahvantia*). This stability motivates the formal definition of the family Hurdiidae, the diagnosis of which comprises the synapomorphies identified in our phylogenetic analysis. The majority of these diagnostic characters pertain to the frontal appendage, for the other aspects of the morphology, such as the number of body segments or the arrangement and shape of carapace elements and trunk flaps, are not consistent across the entire family. The proximal region of the frontal appendage is typically limited to a short peduncle (single podomere?) in most hurdiids, but *Pahvantia* apparently possesses a multisegmented peduncle followed by two pomoderes with well-developed endites (although much shorter than those of the intermediate region).

Lastly, *Schinderhannes* was originally described as a great-appendage arthropod[14], but since then this taxon has been repeatedly retrieved with hurdiid radiodonts in phylogenetic analyses[1,23–25,this study]. Since the morphology of its frontal appendages fits with the diagnosis of the Hurdiidae proposed above, *Schinderhannes* is reassigned to this family and therefore to the Radiodonta.

## Genus *Pahvantia* Robison & Richards[12]

**Diagnosis (emended).** Hurdiid radiodont exhibiting the following unique combination of characters: central element lanceolate (length/width ratio > 2), with a tiny antero-sagittal spine, well-developed lateral lips, a strong postero-lateral constriction, and an indented posterior margin; paired lateral elements tall, with a straight to slightly concave postero-dorsal margin; frontal appendage equipped with at least seven plate-like endites, including two proximal ones bearing at least seven anterior auxiliary spines organised in two rows, and five longer distal ones fringed anteriorly by two rows of 50–60 narrowly-spaced setae.

**Species included.** The type species *Pahvantia hastata* Robison & Richards[12] is the only known representative.

**Remarks.** Robison & Richards[12] provided a short description in place of a diagnosis, which only concerned what is now known to be the central element of a tripartite cephalic carapace. The emended diagnosis proposed above introduces characters related to the lateral elements and frontal appendages, and considers a reverse orientation of the central element (sagittal spine indicates the anterior) compared to the original description.

### *Pahvantia hastata* Robison & Richards[12]

**Holotype.** KUMIP 134878, a small, but relatively complete central element of the cephalic carapace (‘H-element’; Supplementary Fig. 2a).

**Additional specimens.** KUMIP 134187, 134879, 314084, 31490, more or less complete isolated central elements (Supplementary Fig. 2b, c, e, f); KUMIP 155091, isolated lateral element (part and counterpart; Supplementary Fig. 2d); KUMIP 314089, assemblage regrouping a partially dislocated tripartite cephalic carapace and an incomplete frontal appendage (Fig. 1; Supplementary Fig. 2g, h).

**Occurrences.** In the central House Range, at the type locality of the Wheeler Formation in the Wheeler Amphitheater, and two nearby localities in the Antelope Mountain (Supplementary Fig. 1). In the Drum Mountains, at locality MIP-2006-47 (GPS coordinates: 39.509440, -112996890). Western central Utah, USA. See[26] for location details.

**Horizon.** Upper part of the Wheeler Formation, *Ptychagnostus atavus* agnostid biozone, lower Drumian, Miaolingian, Cambrian.

**Diagnosis.** As for genus.

**Description.** Only the morphologies of the cephalic carapace and frontal appendages are known in this taxon. Central element lanceolate, more than twice longer (sag.) than wide (tr.), and terminated anteriorly by a tiny sagittal spine; its median half (antero-posteriorly) is flanked laterally by lateral lips, which are typically well-separated from medial region by abrupt topographical changes; substantially narrower (c. 40 percent of maximum width, tr.) posterior region about a sixth of sclerite length (sag.), and exhibiting three triangular re-entrants (a large central one and two smaller lateral ones) along its posterior margin. Lateral element tall in lateral view, with slightly convex dorsal margin, straight to slightly concave

postero-dorsal margin, and a hook-shaped, anterior process ('beak'); narrow rim apparently running along ventral margin on the internal surface.

Frontal appendage essentially known from its peduncle and the plate-like endites of seven podomeres. Peduncle long and narrow, composed of an uncertain number of podomeres (five?) devoid of endites. Proximalmost two endites short and robust, each bearing seven auxiliary spines on the distal portions of their anterior margins, apparently organised in two rows (Fig. 1f); more distal of the two endites twice as wide as the proximalmost one. More distal (five) endites two to three times wider and about three times longer, all bearing two rows of setae-like structures projecting perpendicularly from their anterior margins (Fig. 1c–e); each row is composed of 50–60 setae, 100–150 µm wide at base, evenly spaced 60–70 µm apart, and arranged subparallel to one another and to setae of other rows; proximal setae (at least) long (up to 3.7 mm), typically overlapping the succeeding (distally) one or two endites (Fig. 1d).

**Remarks.** Although of different sizes, the new dorsal elements and the original specimens[12] are all particularly similar to one another, whether the general outline or details of the morphology (e.g. well-individualized lateral lips, indented posterior margin; Supplementary Fig. 2) are considered. Only the lateral lips might be slightly wider (tr.) relative to the main part of the sclerite in larger specimens, which indicates that the morphology of the dorsal element was subject to limited intra-specific (ontogenetic and/or inter-individual) variations in *P. hastata*. By contrast, the lateral elements of specimen KUMIP314089 exhibit noticeable differences compared to the sole, previously illustrated specimen of this taxon (Supplementary Fig. 2d, h): a more convex dorsal margin, a greater maximum height/maximum length ratio (0.63, instead of 0.49); and a more developed, hook-shaped anterior process. The simpler morphology of this process in specimen KUMIP155091 is

probably due to preservation, but the difference in outline more likely relates to the fact that it is twice as large as the other (KUMIP314089). On the other hand, it has been shown that the shape of the lateral element greatly varies in *Hurdia*, preventing its use for species discrimination[20] – this might also be the case in *Pahvantia*.

Due to incomplete preservation and the superimposition of many elements in the sole specimen available, some aspects of the morphology of the frontal appendage in *P. hastata* remain imperfectly understood. For instance, the peduncle appears abnormally slender for a structure aiming to support to the distal region of the appendage and its large endites – this could be explained by an oblique orientation and/or an incomplete preservation of this proximal region. The morphology of the setae-bearing endites and their apparent branching into two parts distally are even more complicated to understand. Careful examination of the specimen allows a few observations to be made. Firstly, the two parts of a given endite are separated from one another by a slight topographical difference only, and the two of them are located on higher planes than the plane bearing the succeeding (i.e. more distal) endite. This strongly suggests that they do not belong to two separate endites forming a pair, but to a single endite. Secondly, the insertion sites of the setae born by the topographically higher part in the distal region forms a line that approximately follows in the proximal region the mid-width line of the endite (see endite 5 and 6 in Fig. 1c–e); in this proximal region, the setae are long and evidently overlapped what appears as a distinct branch in the distal region. This indicates that the two parts of the endite are already well-expressed proximally, but their arrangement is obscured by the superimposition of the long setae. Lastly, the general orientations of the two parts of a given endite are extremely similar: the two parts of endites 3 exhibit a sharp bent at mid-length; those of endites 4 are also ‘flexed’ at mid-length, but in a smoother way; endite 6 exhibits a more gradual curvature all along and its two parts are orientated subparallel to one another distally, as are those of endite 7. The fact that the two

parts of a given endite are affected the same way by deformation indicates that they are not physically separated (i.e. distinct branches). Based on these observations, we hypothesize that the long endites were relatively thick anteriorly, which allowed the insertion of two rows of setae (Supplementary Fig. 3a). The more difficult preparation of the distal parts of the endites might have led to a partial destruction of the uppermost (as preserved on the fossil) setae, revealing the topographically deeper setae and their insertion sites (Supplementary Fig. 3b). A moderate twisting of the endites distally might have also contributed to the fact that their two rows of setae, separated by thin layers of sediment, appear as distinct branches distally (Supplementary Fig. 3c).

# SUPPLEMENTARY NOTE 2: PHYLOGENETIC ANALYSES

The matrix of characters used for our analyses is available in Lerosey-Aubril & Pates[26].

## LIST OF CHARACTERS

The number in brackets at the end of a character description corresponds to the number assigned to this character, or the character it derives from (when substantially changed), in Vinther et al.[23] (in bold) and Van Roy et al.[1] (in italics).

1. ***GENERAL: Divisions of external body surface*** [**12**]
   - (0) undivided
   - (1) annuli
   - (2) segments

2. ***GENERAL: Digestive glands*** [**16**]
   - (0) absent
   - (1) present

3. ***GENERAL: Paired lateral appendages*** [**17**]
   - (0) absent
   - (1) present

4. ***GENERAL: Number of appendage pairs*** [**19**]
   - (0) 13 or fewer
   - (1) 14 or more

The term ‘appendage’ refers to a limb (including frontal appendage), sclerotized or not, or a flap.

***5. HEAD: Sclerotization*** [**1**]

(0) unsclerotized

(1) bearing one or more sclerotized elements

The terms 'sclerotized elements' refer to sclerotized plates (i.e. anterior sclerite/carapace central element, carapace lateral element, cephalic shield), which excludes sclerotized appendages (e.g. frontal appendages) or appendicular derivatives (e.g. hypostome).

***6. HEAD: Anterior sclerite: size*** [*60*]

(0) Small antero-dorsal plate

(1) Large plate, forming the central element (or 'H-element') of a carapace and projecting beyond frontal appendage anteriorly

Study of Daley & Edgecombe's illustrations[27] suggests that the location of the anterior sclerite in *Anomalocaris canadensis* is not different from that of *A. saron*, *Lyrarapax unguispinus*, and apparently *Amplectobelua symbrachiata* (i.e. it extends forwards beyond the cephalic anterior margin). The anterior sclerite in these taxa is therefore regarded as similar to that of some deuteropods, such as concilitergans, fuxianhuiids or *Odaraia*[28]. In few radiodonts, such as *Aegirocassis*, *Hurdia*, and *Pahvantia*, this plate is much larger (central element or 'H-element') and forms the roof of a chamber extending beyond the frontal appendages anteriorly. In trilobites, like in most crown-group euarthropods, the anterior sclerite is thought to be part of the cephalic shield[28] – accordingly, this character is coded as unknown in *Eoredlichia*.

***7. HEAD: Anterior sclerite: anterolateral margin*** [*59*]

(0) rounded

(1) ogival

(2) forming a forwardly-projecting sagittal spine

Because the anterior sclerite does not represent a distinct plate in trilobites, but was likely integrated into the cephalic shield[28], this character is coded as unknown in *Eoredlichia*.

***8. HEAD: Anterior sclerite: postero-lateral constrictions*** [NEW]

(0) absent

(1) present

The central element of *Pahvantia* exhibits a strong postero-lateral constriction. A similar, but much less pronounced constriction is known in *Hurdia triangulata* and *H. victoria* (fig. 4 in[20], their 'posterior notches'; fig. 4 in[19]). This character is absent in *Aegirocassis* or any other radiodonts with anterior sclerites, as well as in deuteropods with anterior sclerites (e.g. *Chengjiangocaris*).

***9. HEAD: Lateral sclerites*** [*58*]

(0) absent

(1) small plates associated with proximal parts of frontal appendages

(2) large plates (lateral or 'P' elements) forming a cephalic carapace with anterior sclerite

This character refers to the large paired plates flanking laterally the anterior sclerite to form with the latter a large cephalic carapace in *Aegirocassis*, *Hurdia*, and *Pahvantia*. A pair of much smaller lateral sclerites occurs in *Amplectobelua symbrachiata*[8] and *Lyrarapax unguispinus*[25,29]. Whether such lateral sclerites have been integrated into the cephalic shield of deuteropods is unknown, and therefore this character is coded as unknown for *Chengjiangocaris* and *Eoredlichia*.

***10. HEAD: Lateral sclerites: postero-dorsal margin*** [NEW]

(0) convex

(1) straight or concave

The outline of the lateral (‘P’) element is extremely variable in *Hurdia* (fig. 7 in[20]), but its postero-dorsal margin is apparently always either straight or concave. In *Pahvantia,* it is straight or slightly concave too, whereas it is convex in *Aegirocassis*. It is regarded as convex in *Amplectobelua* and *Lyrarapax* too.

***11. HEAD: dorsal sclerites belonging to two or more segments fused into a cephalic shield*** [*61*]

(0) absent

(1) present

This character is coded as present in deuteropods only, for they are the only taxa considered in our analysis to possess a multisegmented head region[28] (see discussion of character 12 below).

***12. HEAD: number of segments*** [**2**]

(0) one

(1) two or more

This character ‘head size’ initially simply meant to reflect the presence/absence of large carapace elements[23]. However, these elements, whether small or large, are all associated with the anteriormost body segment in radiodonts – the head region is actually restricted to this sole segment. The difference between radiodonts with small and large cephalic sclerites only resides in the presence of a small cap-like structure, covering the proximal parts of frontal appendages, or a large chamber-like one (cephalic carapace), enclosing the whole frontal appendages. Some taxa, such as *Anomalocaris*[27] or *Lyrarapax*[24,25,29], exhibit 3–4 narrow segments posterior to the cephalic (protocerebral) one, but this ‘neck region’ is usually regarded as part of the trunk, since its segments are essentially reduced versions of the more posterior trunk ones (e.g. they possess small lateral flaps). Thus, it seems more appropriate to describe the size of the head

region based on the number of segments composing it. This head region is composed of a single segment only for all the taxa considered herein, except the two deuteropods[30].

***13. HEAD: Eyes*** [**3**]

(0) absent

(1) large, composed of clustered visual units (compound)

It has been recently showed that *Kerygmachella* possesses rather large, sessile, compound eyes[31]. Radiodont eyes are typically large and it has been demonstrated in a couple of taxa (i.e. *Anomalocaris*, *Lyrarapax*) that they are made of lenses[24,32]).

***14. HEAD: Eyes: Stalks*** [**4**]

(0) absent

(1) present

***15. HEAD: Mouth: direction*** [**6**]

(0) anterior

(1) ventral

(2) posterior

***16. HEAD: Mouth: circumoral plates*** [**7**]

(0) absent

(1) radially-arranged and sclerotized

*Lyrarapax* species were originally thought to be devoid of oral cone[24,29]. However, it has been recently demonstrated that this genus does possess such a structure, and that its absence in the original material of *L. unguispinus* and *L. trilobus* results from post-mortem disarticulation[25]. The cases of *Amplectobelua symbrachiata*, *Ramskoeldia consimilis*, and *R. platyacantha* are more problematic. Cong et al.[6,8] illustrated the presence of small sclerotized plates, either smooth or tuberculate, in these three taxa, which they interpreted as disarticulated circumoral

plates. Such an easily-disarticulated mouth apparatus would be a significant departure to the condition hitherto described in radiodonts, which are characterized by the great physical integrity of their oral cones (see discussion of character 20 below). Moreover, it is important to keep in mind that the reconstruction proposed by Cong et al.[8] (fig. 10) of the putative mouth apparatus of *A. symbrachiata* is hypothetical (as acknowledged by the authors) – at present, no specimens showing such plates radially organized as a mouth apparatus have been described. Actually, several illustrated specimens display tuberculate plates aligned in series (figs. 1, 5, 8 in[8]; fig. 1 in[6]). In one case, three aligned tuberculate plates are so intimately associated with three, similarly aligned gnathobase-like structures (see discussion of character 20 below) that they look like parts of the same organs (fig. 5 in[8]). These different sclerotized elements are more disorganised in other specimens, but the small and tuberculate plates are both usually found in close association with gnathobase-like structures. On the other hand, the absence of typical radiodont oral cones in the materials of *A. symbrachiata* or any of the two *Ramskoeldia* species illustrated to date is also puzzling and would require further investigations. In summary, it is at present unclear whether the small sclerotized plates of some amplectobeluids truly represent circumoral structures and even much so, if the tuberculate plates, the smooth plates, or both could be homologized with the circumoral teeth of other radiodonts, *Pambdelurion* or some cycloneuralians. Accordingly, all the characters related to the circumoral plates (characters 16–20) have been coded as uncertain for *A. symbrachiata* and *R. consimilis*.

***17. HEAD: Mouth: circumoral plates: size differentiation within main ring*** [NEW]

(0) absent

(1) at least two sizes represented

The mouth apparatus of *Pambdelurion* comprises a main ring composed of single-sized, triangular teeth[33], whereas the main ring of the oral cone of radiodonts is composed of plates

of at least two different sizes. This new character replaces Vinther et al.'s character 8[23] (see discarded characters below). It has been tentatively coded as present in *Anomalocaris kunmingensis*, although this taxon was described using frontal appendages only[34]. This decision is motivated by the discovery of oral cones with tetraradially-arranged large plates from the Guanshan Konservat-Lagerstätte[35]. As explained by these authors, these deposits have yielded a hundred frontal appendages of *A. kunmingensis*, but only one of a second radiodont species (*Paranomalocaris multisegmentalis*). Accordingly, it seems far more probable that the Guanshan oral cones belong to *A. kunmingensis*, especially since a similar oral cone has been described in the closely-related *Lyrarapax unguispinus* since then[25]. Otherwise, this character was coded as uncertain for *A. symbrachiata* and *R. consimilis* (see discussion of character 16 above).

***18. HEAD: Mouth: circumoral plates: distribution of large plates*** [**9**]

(0) triradial
(1) tetraradial

As pointed out by Zeng et al.[35], the oral cones of radiodonts are never, strictly speaking, radially symmetrical structures. In the 'triradial' type (e.g. *Anomalocaris*), the anterior large plate is larger than the two other large plates, and in the 'tetraradial' type (e.g. *Peytoia*), the lateral large plates are larger than the anterior and posterior ones. In other words, the radiodont oral cone is a structure composed of radially arranged plates exhibiting a bilateral symmetry. Accordingly, it seems more appropriate to apply the terms triradial and tetraradial to the way the large plates (whether they are slightly different in size or not) are distributed within the main ring, rather than to the symmetry of the whole organ. Note also that the character states have been reversed compared to Vinther et al.'s character 9[23]. This character was coded as uncertain for *A. symbrachiata* and *R. consimilis* (see discussion of character 16 above).

***19. HEAD: Mouth: circumoral plates: sculpture of plates of main ring*** [**10**]

(0) smooth

(1) bearing rounded or triangular nodes

This character was coded as uncertain for *A. symbrachiata* and *R. consimilis* (see discussion of character 16 above).

***20. HEAD: Mouth: circumoral plates: inner rings of smaller plates forming a cohesive structure with primary ring*** [**11**]

(0) absent

(1) present

The components of the oral cone of radiodonts are reminiscent to the radially-arranged circumoral plates/teeth present in more basal stem-group euarthropods, other panarthropods (e.g. lobopodians), and some cycloneuralians[33,36,37], which suggests a deep origin of this feature in the evolutionary history of ecdysozoans. Yet, the radiodontan oral cone differs from all these other mouth apparatuses by the great cohesion of its elements, which form a single unit. Indeed, radiodontan oral plates are typically found associated together as an oral cone, even when this structure is disarticulated from the body as part of a moult assemblage, or completely isolated following *postmortem* disarticulation and redistribution[19,35,38,39]. This attests to a unique structural integrity of this assemblage of plates, which strikingly contrasts with the situations observed in the non-radiodontan taxa mentioned above, including the relatively closely related *Pambdelurion*, in which the arrangement of plates was apparently easily disturbed after death[33]. This observation extends to the inner rings of small plates characterizing the oral cone of *Hurdia*, which are never found separated from the main plate ring[20]. This is a significant difference compared to the pharyngeal teeth of more basal stem-group euarthropods and cycloneuralians[36], and therefore it is at present unclear whether the two types of structures are

homologous. This character was coded as uncertain for *A. symbrachiata* and *R. consimilis* (see discussion of character 16 above).

***21. HEAD: Protocerebral appendages: sclerotization*** [**23**]

(0) absent

(1) present

The original character 'sclerotization of anterior appendage' was coded as present in deuteropods in previous studies[1,23,24], in reference to the presence of antennae. The latter appendages are now regarded as belonging to a different segment of the body (deutocerebral) compared to the frontal appendages of radiodonts or more basal stem-group euarthropods[24,30], which means that the original character referred to non-homologous structures. We rephrase the character definition, so as to restrict it to the appendages associated with the anteriormost (protocerebral) segment of the head. This redefined character remains coded as present for the two deuteropods, but this time in reference to the presence of a hypostome/labrum complex (see character 22).

***22. <u>HEAD: Protocerebral appendages: type</u>*** [NEW – replacing *18, 62*]

(0) Frontal appendages made of annuli

(1) Frontal appendages made of segments

(2) Appendages fused into a hypostome/labrum

It has been demonstrated that the anteriormost pair of appendages in onychophorans, gilled-lobopodians, and radiodonts is/was innervated by the protocerebrum[24,31], unlike that of deuteropods. In the latter organisms, the anteriormost pair of functional appendages are antenniform or chelate and innervated by the deutocerebrum (see character 46), and it is thought that the protocerebral appendages have evolved into the hypostome-labrum complex[30]. Our new character aims to describe this critical difference between basal stem-euarthropods

and radiodonts on the one hand, and deuteropods on the other. It replaces character 18 ('frontal limb structure') and 62 ('labrum') of Van Roy et al.[1].

***23. HEAD: Frontal appendages (FA): orientation of terminal spine/claw*** [**28**]

(0) terminal spine hooked downwards

(1) terminal spine hooked upwards

***24. HEAD: FA: Dorsal spines*** [**29**]

(0) absent

(1) present

***25. HEAD: FA: Endites*** [**25**]

(0) absent

(1) present

***26. HEAD: FA: Endites: length*** [**31**]

(0) short

(1) at least two exceed in length the heights of the podomeres bearing them

This character is coded as absent in amplectobeluids, for they possess only one hypertrophied endite, which exceeds in length the height of the podomere bearing it. It is coded as present in hurdiids and tamisiocaridids (see character 30 below).

***27. HEAD: FA: Endites: width*** [**33**]

(0) strongly narrowing distally (spiniform)

(1) remaining wide distally (plate-like)

***28. HEAD: FA: Endites: width evolution towards appendage distal tip*** [**34**]

(0) remains unchanged or decreases

(1) increases

***29. HEAD: FA: Endites: orientation of proximal endites*** [**35**]

(0) project ventrally

(1) project anteriorly (at least 45 degrees) to oppose tip of appendage, forming a claw

***30. HEAD: FA: Endites: single hypertrophied endite*** [**36**]

(0) absent

(1) present

The terms ‘hypertrophied endite’ refer to an endite that is at least twice as wide at its base as any other endites of the same appendage. This character aims to describe the morphological differentiation of a single endite characterising the family Amplectobeluidae. As such, it differs from character 26 (see above), which relates to the differentiation of the whole series of endites and the discrimination of three main functional types of frontal appendages: grasping, filtering, sediment-sifting[7,40].

***31. HEAD: FA: Endites: individual curvature*** [**39**]

(0) straight or slightly curved

(1) curved anteriorly, talon-like

(2) strongly curved anteriorly, tip at 90 degree with base

***32. HEAD: FA: Endites: curvature variability*** [**46**]

(0) all are straight or curved anteriorly

(1) all are curved posteriorly

(2) only proximal ones are curved posteriorly

***33. HEAD: FA: Endites: length of adjacent endites*** [**44**]

(0) roughly similar

(1) alternating on even and odd podomeres

***34. HEAD: FA: Endites: lengths of adaxial and abaxial (relative to body sagittal axis) endites of a given pair*** [**45**]

(0) subequal

(1) adaxial endite reduced relative to abaxial one or absent

This character was coded as inapplicable for *Aysheia* and *Kerygmachaela,* for their (soft) frontal appendages apparently bear a single row of endites each[31,41,42].

***35. HEAD: FA: Endites: Auxiliary spines*** [**37**]

(0) absent

(1) present

***36. HEAD: FA: Endites: Anterior setae*** [NEW]

(0) absent

(1) present

Van Roy et al.[1] demonstrated the presence of setae inserting within sockets on the anterior margins of endites in *Aegirocassis benmoulae.* The particularity is described by a new character, rather than a new state of character 37, for setae and spines are radically different cuticular structures in arthropods, which can be regarded as homologous in rare instances only[43]. The filtering structures of *Pahvantia* are tentatively interpreted as setae, for they are considerably much thinner and much longer than the auxiliary spines born by the two more proximal endites. Yet, we fully acknowledge the fact that this interpretation will remain tentative until it can be shown that these structures were articulated with the general cuticle or at least, that they have circular bases[44]. To be consistent with our description of *Pahvantia,* we coded this character as present in this taxon, but an attempt at coding it as absent revealed no impact on the phylogenetic position of this taxon, including with regard to *Aegirocassis*.

***37. HEAD: FA: Endites: Auxiliary spines: serial distribution*** [NEW]

(0) auxiliary spines on the endites of several podomeres

(1) auxiliary spines on the endites of a single podomere only

***38. HEAD: FA: Endites: Auxiliary spines: arrangement*** [**38**]

(0) radiating

(1) pectinate

This character was kept, but it should be noted that the differentiation between the two types of arrangement is only obvious in taxa with numerous auxiliary spines. It was coded as inapplicable for *Anomalocaris canadensis* and *Caryosyntrips serratus* – the former possesses a single auxiliary spine per margin, the latter lacks auxiliary spines.

***39. HEAD: FA: Endites: Auxiliary spines: modal number of anterior ones*** [**40**]

(0) none

(1) one

(2) two

(3) three

(4) four or more

***40. HEAD: FA: Endites: Auxiliary spines: orientation of anterior ones*** [**41**]

(0) projecting distally, towards tip of endites

(1) projecting anteriorly, towards tip of appendage

***41. HEAD: FA: Endites: Auxiliary spines: posterior one(s)*** [**42**]

(0) absent

(1) present on one or more endites

***42. HEAD: FA: Endites: Auxiliary spines: number of posterior one(s)*** [**43**]

(0) one

(1) two or more

*Amplectobelua symbrachiata* has always been described as having only one auxiliary spine on each margin of its hypertrophied endite (e.g.[8]), but some specimens clearly show the presence of two posterior auxiliary spines – one is particularly large and the other much smaller and more proximally located (see fig. 19.5, 19.6 in[44]; supplementary fig. 3A in[25]). This small intraspecific variation is probably related to ontogeny, as 1) the hitherto unnoticed auxiliary spine is particularly obvious in large specimens and 2) the addition of auxiliary spines is one of the rare ontogenetic changes reported in radiodonts[25,45].

***43. HEAD: FA: total number of podomeres*** [**24**]

(0) 14 or more

(1) 13 or fewer

This character was coded as inapplicable for taxa with non-sclerotized frontal appendages (e.g. gilled lobopodians) and taxa with a hypostome-labrum complex (i.e. deuteropods; see character 22 above).

***44. HEAD: FA: length of distal podomeres*** [**26**]

(0) elongate

(1) short and closely packed relative to proximal podomeres

This character was coded as inapplicable for taxa with non-sclerotized frontal appendages (e.g. gilled lobopodians) and taxa with a hypostome-labrum complex (i.e. deuteropods; see character 22 above).

***45. HEAD: FA: curvature*** [**27**]

(0) straight or down curved proximally

(1) with a dorsal kink, separating a proximal peduncle (or shaft) from a distal more flexible part (fully articulated podomeres)

This character was coded as inapplicable for taxa with non-sclerotized frontal appendages (e.g. gilled lobopodians) and taxa with a hypostome-labrum complex (i.e. deuteropods; see character 22 above).

***46. <u>HEAD/TRUNK: Deutocerebral appendages</u>*** [NEW – replacing **22**]

(0) absent or undifferentiated compared to more posterior appendages

(1) differentiated compared to more posterior appendages, either antenniform or chelate

Some radiodonts, such as *Amplectobelua symbrachiata*[8], *Ramskoeldia consimilis* and *R. platyacantha*[6], possess sclerotized trunk structures known as gnathobase-like structures associated with the segment immediately following the monosegmented head region posteriorly. In other words, there is evidence for the presence of partially sclerotized appendages in the anterior trunk, including a pair associated with the deutocerebral segment and therefore likely homologous to the deutocerebral appendages of deuteropods. However, as emphasized by Ortega-Hernández[30], the deutocerebral appendages are morphologically differentiated from all the other appendages of the body in all deuteropods, being either antenniform or chelate. This new character aims to describe this fundamental difference between deuteropods and basal stem-euarthropods. It was coded as absent for all radiodonts known from body specimens, and as uncertain for the ones known from frontal appendages only.

***47. TRUNK: shape*** [**13**]

(0) cylindrical

(1) broad anteriorly and tapering posteriorly, maximum width of trunk anteriorly about 300% width of posteriormost trunk segment

***48. TRUNK: arthrodisation*** [**15**]

(0) absent

(1) present

The recently described gnathobase-like structures of some radiodonts[6,8] (see character 46) attest to the presence of a partial sclerotization of the trunk region in some radiodont taxa. The character was rephrased to exclusively refer to the evolution of sclerotized body segments (arthrodisation) in the trunk region, a character as-yet unknown in radiodonts.

***49. TRUNK: relative length of segments*** [**14**]

(0) segments subequal along length of body

(1) posterior segments reduced, with anterior segments 150% or more the lengths of posterior ones

***50. TRUNK: Appendages: sclerotisation*** [**48**]

(0) absent

(1) present

***51. TRUNK: Appendages: ventral element*** [*49*]

(0) independent annulated limb (lobopodous limb)

(1) independent ventral flap

(2) segmented ramus, either independent or attached to a dorsal element to form a biramous appendage

In the overwhelming majority of deuteropods, the ventral and dorsal elements (endopod and exopod, respectively) are segmented rami fused into a biramous appendage. In rare taxa, such as *Dibasterium*[46] and *Habelia*[47], these two branches apparently insert separately on the body.

***52. TRUNK: Appendages: dorsal element*** [**20**, *21*]

(0) absent

(1) independent dorsal flap

(2) segmented ramus, either independent or attached to a ventral element to form a biramous appendage

***53. TRUNK: Appendages: dorsal element: exites/setal blades*** [*20*]

(0) absent

(1) present

***54. TRUNK: Appendages: ventral and dorsal elements fused into a biramous appendage*** [**57**]

(0) absent

(1) present

***55. TRUNK: Dorsal flaps: length*** [*50*]

(0) subequal along length of body

(1) anterior flaps about twice as long (exsagittally) as posterior flaps

***56. TRUNK: Dorsal flaps: internal reinforcements*** [**52**, *53*]

(0) absent

(1) present

The terms ‘internal reinforcements’ refer to the structures previously described as ‘strengthening rays’, ‘transverse lines’ or ‘veins’[29].

***57. TRUNK: Exites/setal blades: distribution*** [**51**, *51*]

(0) confined to lateral region (dorsal flaps or exopods)

(1) extending to median region of the body dorsally

***58. TRUNK: Exites/setal blades: fusion*** [*52*]

(0) absent

(1) present (medio-dorsally)

***59. TRUNK: Tailfan*** [**53**, *54*]

(0) absent

(1) present

This character was coded as present in the deuteropod *Chengjiangocaris*, for this taxon possesses a pair of fringed caudal flaps (fig. 4 in[48]).

***60. TRUNK: Tailfan: type*** [*55*]

(0) small fluke (single pair of flaps)

(1) large fan (multiple pairs of flaps)

***61. TRUNK: Furca*** [**54**, *56*]

(0) absent

(1) present

This character was coded as uncertain for *Aegirocassis*, because the mode of preservation of all known body specimens of this taxon is atypical for the Fezouata or any lower Palaeozoic Burgess Shale-type Konservat-Lagerstätten[49]. This might have had an impact on the ability to preserve appendicular derivatives (including furcal rami), as suggested by the fact that none of these body fossils are associated with frontal appendages. Moreover, a single specimen of *Aegirocassis* exhibits the putative posterior extremity of the trunk and this part of the fossil is poorly preserved (figure S2 in[50]).

## DISCARDED CHARACTERS

**Character 5 (preoral scleral plate)**

Although both belonging to the protocerebral segment, the anterior sclerite and hypostome-labrum complex represent two distinct structures, which may co-occur in some taxa (e.g. fuxianhuiids[51]. The hypostome-labrum complex of deuteropods likely evolved from the fusion of a pair of protocerebral appendages, and therefore is better regarded as homologous to the frontal appendages of radiodonts, rather than their anterior sclerite[28,30]. The presence of these distinct features is now considered separately via characters 5 and 22.

**Character 8 (overlapping of mouth plates)**

This character was initially proposed to distinguish between the radiodont oral cone, and the supposedly more loosely organised mouth plates of *Pambdelurion*[23]. However, it was recently showed that the mouth apparatus of this taxon is rather complex and well-organised[33], and it seems that the overlapping of the plates may not be the most appropriate character to discriminate the conditions observed in *Pambdelurion* and in radiodonts. We propose a new character instead (Character 17), which relates to the presence/absence of different plate sizes in the main ring of plates.

**Character 22 (antennae)**

This character has been replaced by a more precise character describing the presence of deutocerebral appendages differentiated from more posterior appendages in deuteropods.

**Character 30 (dorsal spines: shape)**

This character has proved virtually impossible to code, for the 'short and robust' and 'long and needle-like' types of Vinther et al.[23] actually represent extreme morphs of a continuum.

**Character 32 (longest endite on podomere 2)**

There is already a character related to the presence of a single pair of hypertrophied endite. Moreover, determining to which podomere it belongs to is in practise rarely possible, due to incomplete preservation of the proximalmost part of the appendage in many taxa and when a peduncle is differentiated, the effacement of podomere boundaries in this region (see characters not selected below).

## CHARACTERS CONSIDERED, BUT NOT SELECTED

**Presence/absence of an anterior sclerite**

With the present selection of taxa, this character appeared redundant with character 5 (head sclerotization). Indeed, character 5 excludes the sclerotization of frontal appendages, which in practise means that it refers to the presence of an anterior sclerite (possibly integrated in a cephalic shield in deuteropods), for none of the taxa considered possess lateral elements only. If such a taxon is discovered in the future, the presence/absence of an anterior sclerite would be a valid additional character to consider.

**Number of podomeres composing the peduncle of the frontal appendage**

According to Cong et al.[6], a frontal appendage with a three-segmented peduncle (their shaft) is a diagnostic character of the family Amplectobeluidae, other radiodonts having a two-segmented peduncle (e.g. *Anomalocaris*) or no differentiated peduncle whatsoever. However, we believe that the number of podomeres composing the peduncular region is a character too problematic to be used in a phylogenetic analysis of the Radiodonta, at least for now. Firstly, the proximalmost region of most specimens of frontal appendages is either incomplete or concealed under the head, which prevents the determination of the number of podomeres composing the peduncle (when present) in most taxa. Secondly, the peduncle appears straighter compared to the more distal *articulated* region and has less pronounced podomere boundaries (articulation ankylosis?). This is demonstrated for example in amplectobeluids, where podomere boundaries in the peduncle are much harder to discern than the ones occurring in the distal, flexible region (e.g. extended data fig. 1b in[24]; figs. 2, 5, 6 in[8]; see also[52]). Thirdly – and this might be related to the preceding observation or not – evidence for the presence of three podomeres in the peduncle of amplectobeluids is scarce. Careful examination by one of us (RLA) of large appendages of *Amplectobelua symbrachiata* failed to discern more than two

peduncular podomeres – a long, enditeless podomere 1 with a proximal margin broadly convex, except for a small notch (i.e. somewhat reminiscent to that of *Anomalocaris briggsi* [39]), and an at least twice shorter podomere 2 with a simple endite abutting the ventral margin of the first podomere of the distal articulated region (i.e. the one bearing the hypertrophied endite). The only specimen illustrating the three-segmented peduncle of *A. symbrachiata* is the holotype (fig. 4 in[8]); however, the part of the fossil interpreted as podomere 1 exhibits a dense annulation, rather than being smooth, and it forms a sharp angle with the rest of the peduncle, a feature not observed in any other specimens, where the peduncle is stiff and straight. In the frontal appendages of *Ramskoeldia consimilis* and *R. platyacantha* illustrated[33], the segmentation of the peduncle is either totally absent or hardly discernible. To sum up, whether the number of peduncular podomeres is different in amplectobeluids remains at best uncertain, and even if it is, preservation rarely allows a confident count of the number of peduncular podomeres to be made in most peduncle-bearing radiodonts, which considerably limits the use of this character in cladistics.

**Presence of gnathobase-like structures (GLS)**

Chen et al.[52] first described the presence of rectangular sclerotized elements with a strongly spinose margin in *Amplectobelua symbrachiata* and interpreted them as components of a mouth apparatus ('teeth'). Recently, this view was challenged by Cong et al.[8], who convincingly showed that these elements were aligned along an exsagittal axis and apparently associated with the reduced lateral flaps of the anterior trunk region (or neck region[29]). Since then, similar structures have been described in species of *Ramskoeldia*[6], which suggests that they might be more common in radiodonts and therefore phylogenetically informative within this group. Moreover, gnathobases are present in a great diversity of Cambrian euarthropods (e.g. habellids[47]; nektaspidids[53]; sanctacaridids[54]; trilobites[55]; *Sidneyia*[56]). Along with their

recent description in fuxianhuiids[48], this suggests that gnathobases might have a deep origin in deuteropods, which invites comparison with the structures described in some radiodonts. Yet, we refrained from introducing a new character referring to GLS/gnathobases, because of the uncertain nature of radiodontan GLS. Indeed, similarities with onychophoran jaws indicate that they might represent distal, rather than proximal parts of appendages[8].

## NEW TAXA

The recently described *Laminocaris chimera*[7] and *Ramskoeldia consimilis*[6] were added to increase the generic diversity (and morphological disparity) of radiodonts represented by the selection of taxa considered in our phylogenetic analysis. An attempt at introducing two recently described species of *Caryosyntrips*[57], along with a character 'ankylosis of all podomere articulations' restricted to them, did not result in the recovery of a monophyletic clade Radiodonta including this genus or even a monophyletic clade *Caryosyntrips*. The internal relationships within the genus and the relationships of the three species with other radiodonts and deuteropods were unresolved, leading us to ultimately keep one representative of the genus only.

## TAXA REPLACED

The middle Cambrian *Olenoides serratus* is unique among trilobites in possessing a pair of cerci (paired, antenniform appendages inserted on the posteriormost body segment) and digestive glands associated with pygidial segments[58,59]. Accordingly, it has been replaced by the early Cambrian *Eoredlichia intermedia*, considered as more representative of the morphology of early representatives of the Trilobita.

## TAXA DISCARDED

Three taxa considered by Van Roy et al.[1] have proved inadequate for investigating the phylogenetic relationships within the Radiodonta or between this group and more stemward/crownward euarthropods: *Anomalocaris* cf. *saron* (Pioche Formation), *Anomalocaris pennsylvanica*, *Cucumericrus decoratus*. These taxa are each represented by a few poorly preserved specimens, leading to code most of the characters of our analysis as uncertain for them (e.g. 53 of the 61 characters in the case of *C. decoratus*). Running the analysis with them resulted in: 1) retrieving these taxa in unresolved positions, in polytomy with *Caryosyntrips*, Deuteropods and radiodonts for *A. pennsylvanica* and *C. decoratus* and with amplectobeluids for *A.* cf. *saron* (Pioche Formation); 2) recovering two to three times more most-parsimonious-trees (MPTs). Conversely, removing them considerably reduced the number of MPTs recovered, while modifying nothing to the topology of the rest of the tree. In other words, the inclusion of these poorly known taxa does not permit to increase the resolution or stability of the trees recovered, hence the reason why we ultimately discarded them in our analysis.

# SUPPLEMENTARY FIGURES

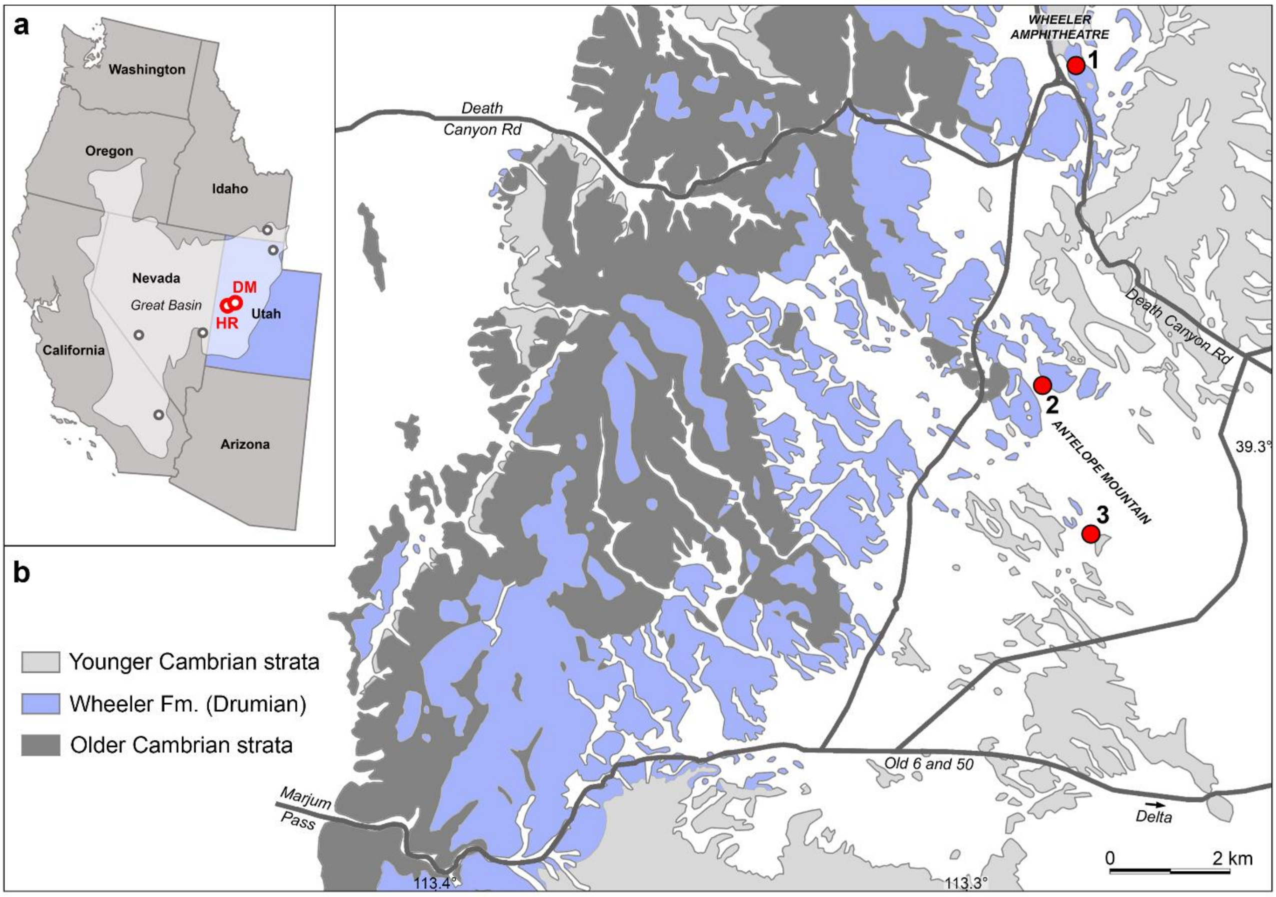


**Supplementary Fig. 1. Exposures of the Drumian (Miaolingian) Wheeler Formation yielding *Pahvantia hastata*. a** Map of western USA showing the locations of the Cambrian Konservat-Lagerstätten (circles) of the Great Basin (white area). *P. hastata* is found the Wheeler Formation in both the House Range (HR) and the Drum Mountains (DM), western Utah. **b** Simplified geological map of the central House Range, showing the geographic distribution of the Wheeler Formation and the locations of the three outcrops yielding *P. hastata* (red dots). Data relative to the spatial distributions of Cambrian strata are derived from[60].

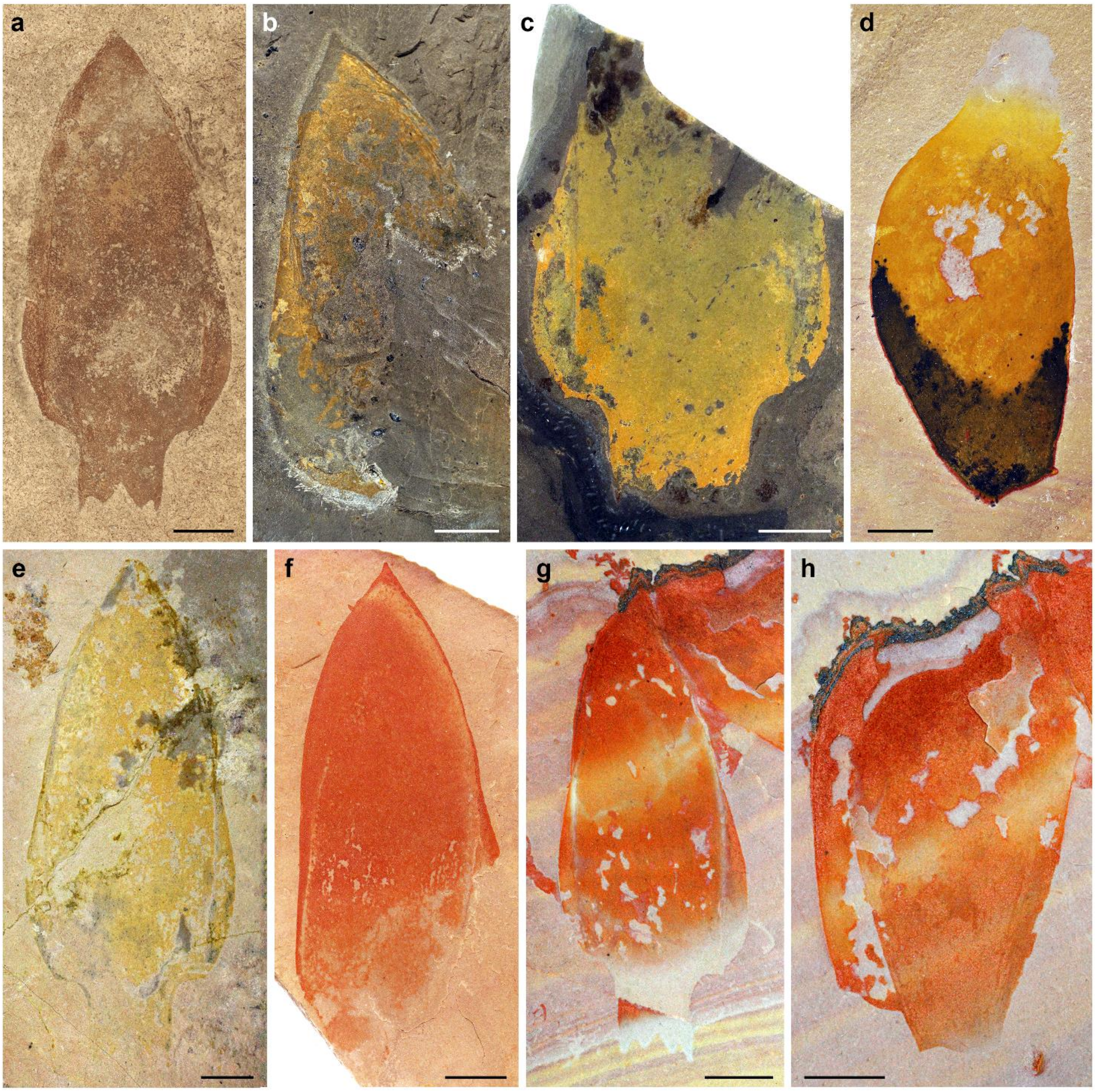


**Supplementary Fig. 2. Cephalic carapace elements of *Pahvantia hastata*.** Photographs of specimens dry (**b**, **d**, **e**) or immersed in dilute ethanol (**a**, **c**, **f–h**). **a**–**c**, **e**–**g** central elements of the cephalic carapace in dorsal views, with anterior end facing to the top. **a** holotype KUMIP134878. **b** topotype KUMIP134187. **c** topotype KUMIP134879. **e** KUMIP314084 (sole specimen from the Drum Mountains). **f** KUMIP314090. **g** KUMIP314089. **d**, **h** lateral elements of the cephalic carapace in lateral views, with anterior end facing to the top. **d** KUMIP155091. **h** KUMIP314089. Scale bars represent 1 cm (**b**–**d**) and 5 mm (**a**, **e**–**h**).

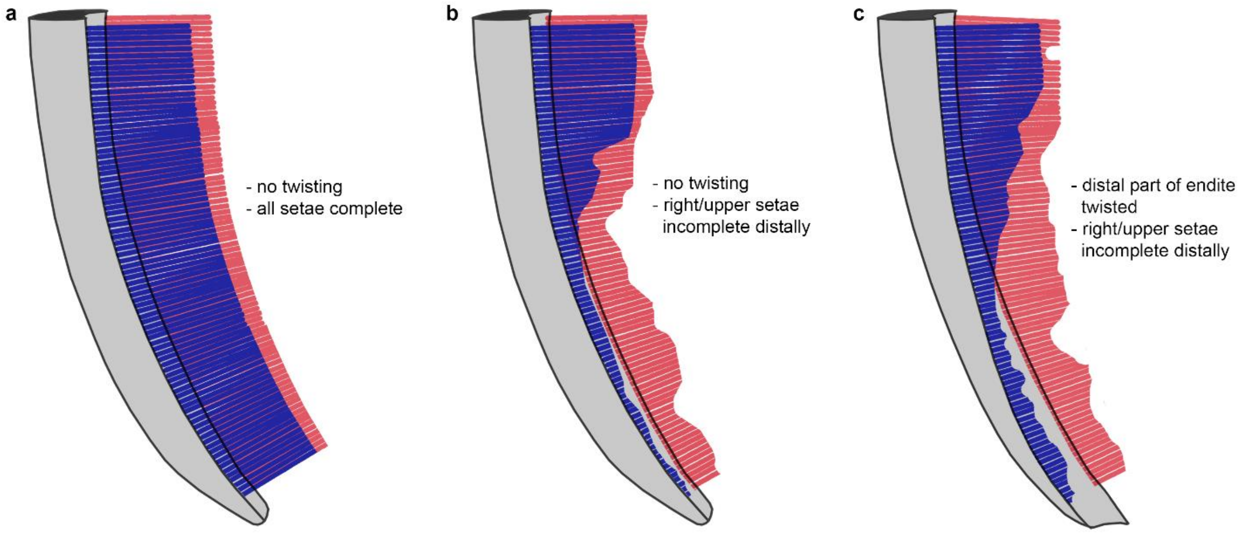


**Supplementary Fig. 3. Taphonomic scenario explaining in the observed morphology of the frontal appendage of *Pahvantia hastata*.**

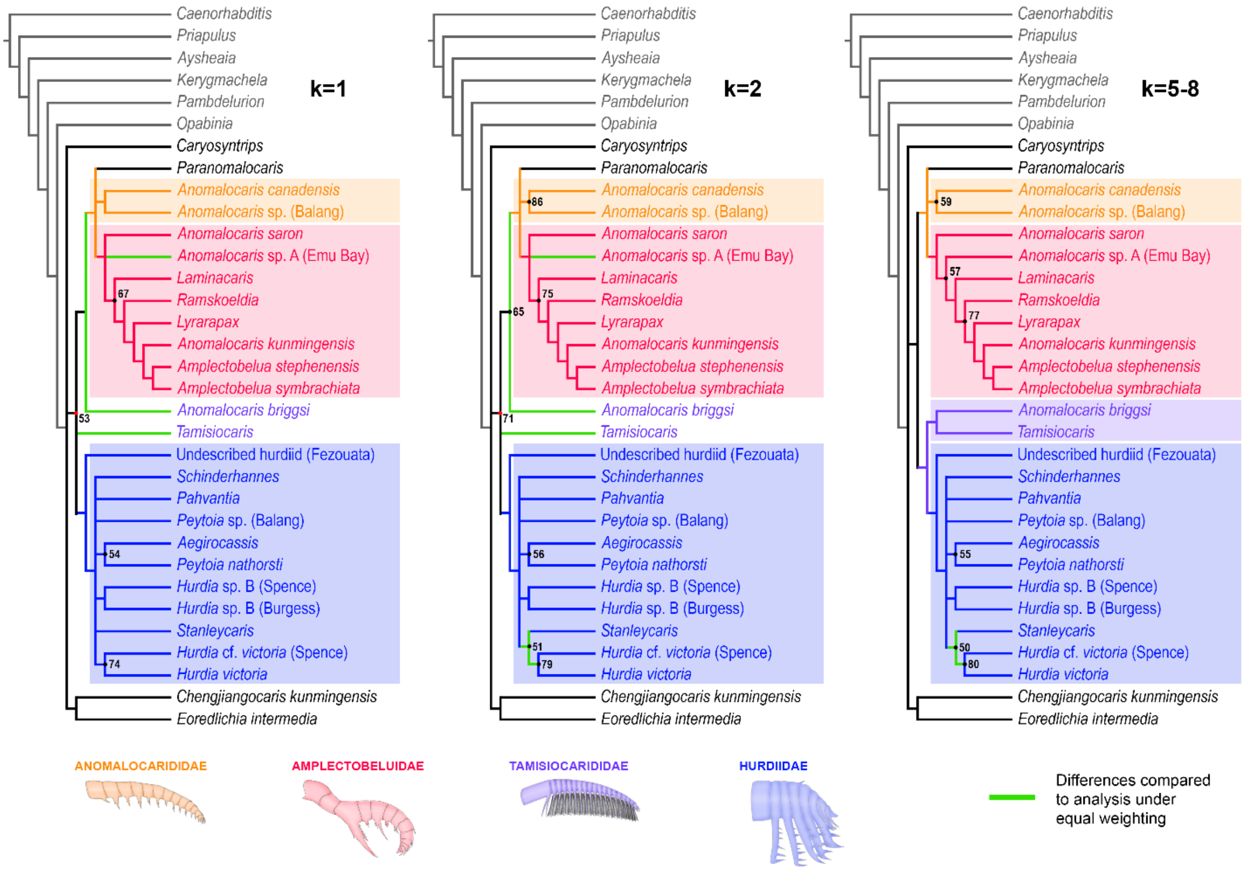


**Supplementary Fig. 4. Variations of tree topology of parsimony analyses under implied weighting and various values of concavity constant k.** Except for the paraphyly of Tamisiocarididae, retrieved with k values of 1 and 2 only, changes to tree topology compared to that obtained under equal weighting are extremely minor. Tree topologies retrieved with k values of 3, 4, 9, and 10 are not represented, for they are strictly identical to that obtained under equal weighting.

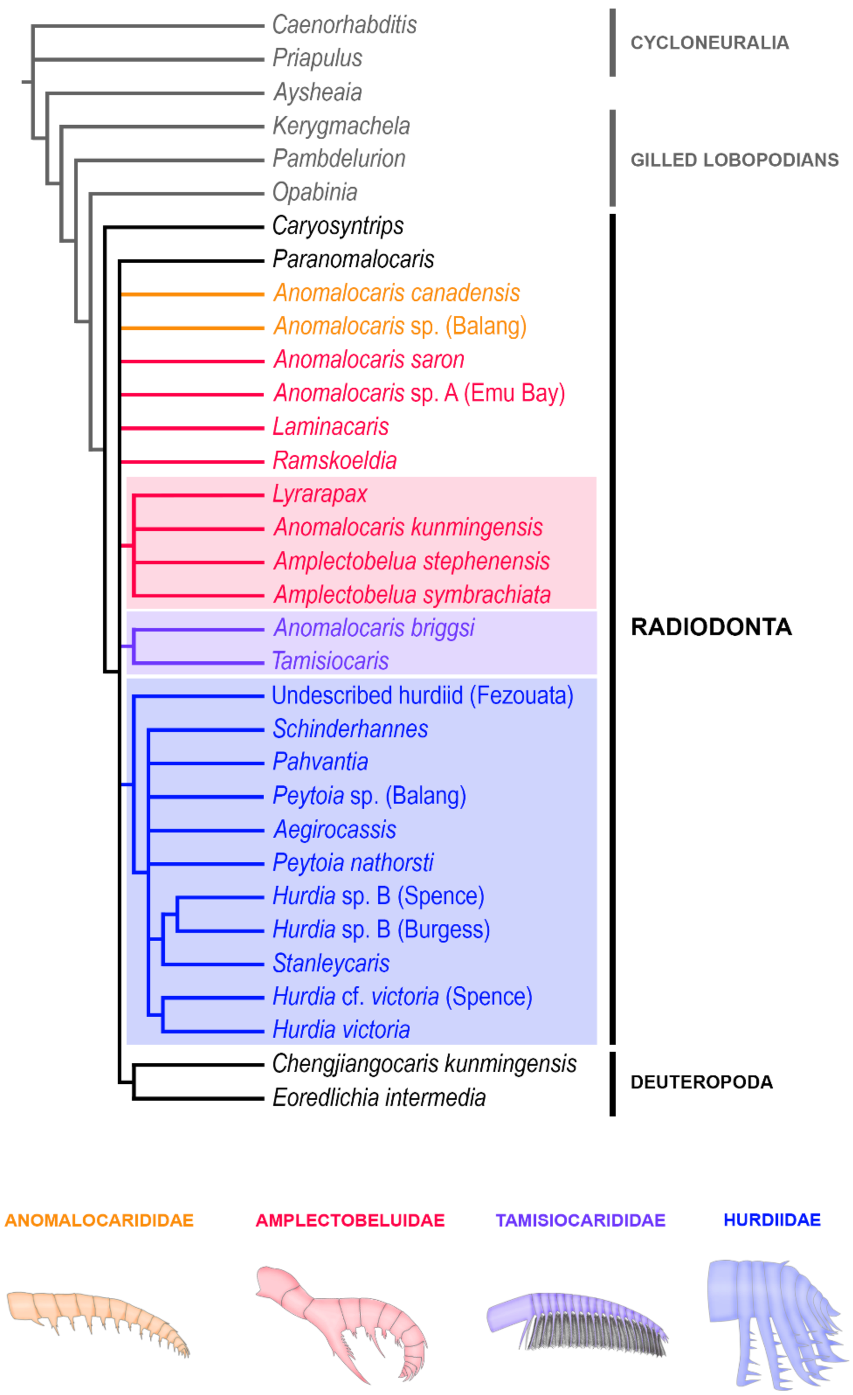


**Supplementary Fig. 5. Results of the Bayesian inference analysis.** Despite a poor resolution of the phylogenetic relationships within the Radiodonta, *Pahvantia* and *Aegirocassis* are retrieved within a monophyletic family Hurdiidae, which is distinct from the monophyletic family Tamisiocarididae. This indicates that suspension-feeding evolved at least twice in the history of radiodonts.

# SUPPLEMENTARY REFERENCES


1. Van Roy, P., Daley, A. C. & Briggs, D. E. G. Anomalocaridid trunk limb homology revealed by a giant filter-feeder with paired flaps. *Nature* **522,** 77–80 (2015).
2. Daley, A. C., Budd, G. E., Caron, J.-B., Edgecombe, G. D. & Collins, D. The Burgess Shale anomalocaridid *Hurdia* and its significance for early euarthropod evolution. *Science* **323**, 1597–1600 (2009).
3. Chen, J., Waloszek, D. & Maas, A. A new 'great-appendage'arthropod from the Lower Cambrian of China and homology of chelicerate chelicerae and raptorial antero-ventral appendages. *Lethaia* **37**, 3–20 (2004).
4. Haug, J. T., Waloszek, D., Maas, A., Liu, Y. & Haug, C. Functional morphology, ontogeny and evolution of mantis shrimp-like predators in the Cambrian. *Palaeontology* **55**, 369–399 (2012).
5. Hou, X., Bergström, J. & Ahlberg, P. Anomalocaris and other large animals in the lower Cambrian Chengjiang Fauna of southwest China. *GFF* **117**, 163–183 (1995).
6. Cong, P., Edgecombe, G. D., Daley, A. C., Guo, J., Pates, S. & Hou, X. G. New radiodonts with gnathobase-like structures from the Cambrian Chengjiang Biota and implications for the systematics of Radiodonta. *Pap. Palaeontol.* https://doi.org/10.1002/spp2.1219 (2018).
7. Guo, J., Pates, S., Cong, P., Daley, A. C., Edgecombe, G. D., Chen, T. & Hou, X. A new radiodont (stem Euarthropoda) frontal appendage with a mosaic of characters from the Cambrian (Series 2 Stage 3) Chengjiang biota. *Pap. Paleontol.* https://doi.org/10.1002/spp2.1231 (2018).

8. Cong, P., Daley, A. C., Edgecombe, G. D. & Hou, X. G. The functional head of the Cambrian radiodontan (stem-group Euarthropoda) *Amplectobelua symbrachiata*. *BMC Evol. Biol.* **17**, 208 (2017).
9. Briggs, D.E.G. *Anomalocaris*, the largest known Cambrian arthropod. *Palaeontology* **22**, 631–664 (1979).
10. Collins, D. The "evolution" of *Anomalocaris* and its classification in the arthropod class Dinocarida (nov.) and order Radiodonta (nov.). *J. Paleontol.* **70**, 280–293 (1996).
11. Walcott, C. D. Middle Cambrian Branchiopoda, Malacostraca, Trilobita and Merostomata. *Smithsonian Miscel. Collect.* **57**, 145–228 (1912).
12. Robison, R. A. & Richards, B. C. Larger bivalve arthropods from the middle Cambrian of Utah. *Univ. Kansas Paleontol. Contr.* **106**, 1–28 (1981).
13. Walcott, C. D. Cambrian Geology and Paleontology II. Middle Cambrian holothurians and medusae. *Smithsonian Miscel. Collect.* **57**, 41–68 (1911).
14. Kühl, G., Briggs, D. E. G. & Rust, J. A great-appendage arthropod with a radial mouth from the Lower Devonian Hunsrück Slate, Germany. *Science* **47**, 771-773 (2009).
15. Pates, S., Daley, A. C. & Ortega-Hernández, J. Reply to Comment on "*Aysheaia prolata* from the Utah Wheeler Formation (Drumian, Cambrian) is a frontal appendage of the radiodontan *Stanleycaris*" with the formal description of *Stanleycaris*. *Acta Palaeontol. Pol.* **63**, 105–110 (2018).
16. Daley, A. C. & Legg, D. A. A morphological and taxonomic appraisal of the oldest anomalocaridid from the Lower Cambrian of Poland. *Geol. Mag.* **152**, 949–955 (2015).
17. Liu, Q. The first discovery of anomalocaridid appendages from the Balang Formation (Cambrian Series 2) in Hunan, China. *Alcheringa* **37**, 1–6 (2013).
18. Cui, Z.-l. & Huo, S.-c. New discoveries of Lower Cambrian crustacean fossils from western Hubei. *Acta Palaeontol. Sin.* **29**, 321–330 (1990).

19. Pates, S., Daley, A. C. & Lieberman, B. S. Hurdiid radiodontans from the middle Cambrian (Series 3) of Utah. *J. Paleontol.* **92**, 99–113 (2018).

20. Daley, A. C., Budd, G. E. & Caron, J.-B. Morphology and systematics of the anomalocaridid arthropod *Hurdia* from the middle Cambrian of British Columbia and Utah. *J. Syst. Palaeontol.* **11**, 743–787 (2013).

21. Chlupáč, I. & Kordule, V. Arthropods of Burgess Shale type from the Middle Cambrian of Bohemia (Czech Republic). *Bull. Czech Geol. Surv.* **77**, 167–182 (2002).

22. Masiak, M. & Zylińska, A. Burgess Shale-type fossils in Cambrian sandstones of the Holy Cross Mountains. *Acta Palaeontol. Pol.* **39**, 329–340 (1994).

23. Vinther, J., Stein, M., Longrich, N. R. & Harper, D. A. T. A suspension-feeding anomalocarid from the Early Cambrian. *Nature* **507,** 496–499 (2014).

24. Cong, P., Ma, X., Hou, X., Edgecombe, G. D. & Strausfield, N. J. Brain structure resolves the segmental affinity of anomalocaridid appendages. *Nature* **513**, 538-542 (2014).

25. Liu, J. Lerosey-Aubril, R., Steiner, M., Dunlop, J. A., Shu, D. & Paterson, J. R. Origin of raptorial feeding in juvenile euarthropods revealed by a Cambrian radiodontan. *Nat. Sci. Rev.* https://doi.org/10.1093/nsr/nwy057 (2018).

26. Lerosey-Aubril, R. & Pates, S. Data from: New suspension-feeding radiodont suggests evolution of microplanktivory in Cambrian macronekton. Dryad Digital Repository. doi:10.5061/dryad.1cf2fb0 (2018).

27. Daley, A. C. & Edgecombe, G. D. Morphology of *Anomalocaris canadensis* from the Burgess Shale. *J. Paleontol.* **88**, 68–91 (2014).

28. Ortega-Hernández, J. Homology of head sclerites in Burgess Shale euarthropods. *Curr. Biol.* **25**, 1625–1631 (2015).

29. Cong, P., Daley, A. C., Edgecombe, G. D., Hou, X. & Chen, A. Morphology of the radiodontan *Lyrarapax* from the early Cambrian Chengjiang biota. *J. Paleontol.* **90,** 663–671 (2016).

30. Ortega-Hernández, J. Making sense of 'lower' and 'upper' stem-group Euarthropoda, with comments on the strict use of the name Arthropoda von Siebold, 1848. *Biol. Rev.* **91**, 255–273 (2016).

31. Park, T.-Y. S., Kihm, J.-H., Woo, J., Park, C., Lee, W. Y., Smith, M. P., Harper, D. A. T., Young, F., Nielsen, A. T. & Vinther, J. Brain and eyes of *Kerygmachela* reveal protocerebral ancestry of the panarthropod head. *Nat. Comm.* **9**, 1019 (2018).

32. Paterson, J. R., García-Bellido, D. C., Lee, M. S. Y., Brock, G. A., Jago, J. B. & Edgecombe, G. D. Acute vision in the giant Cambrian predator *Anomalocaris* and the origin of compound eyes. *Nature* **480**, 237–240 (2011).

33. Vinther, J., Porras, L., Young, F. J., Budd, G. E. & Edgecombe, G. D. The mouth apparatus of the Cambrian gilled lobopodian *Pambdelurion whittingtoni*. *Palaeontology* **59**, 841–849 (2016).

34. Wang, Y., Huang, D. & Hu, S. New anomalocaridid frontal appendages from the Guanshan biota, eastern Yunnan. *Chin. Sci. Bull.* **58**, 3937–3942 (2013).

35. Zeng, H., Zhao, F., Yin, Z. & Zhu, M. A new radiodontan oral cone with a unique combination of anatomical features from the early Cambrian Guanshan Lagerstätte, eastern Yunnan, South China. *J. Paleontol.* **92**, 40–48 (2018).

36. Vannier, J., Liu, J., Lerosey-Aubril, R., Vinther, J. & Daley, A. C. Sophisticated digestive systems in early arthropods. *Nat. Comm.* **5**, 3641 (2014).

37. Smith, M. R. & Caron, J.-B. *Hallucigenia*'s head and the pharyngeal armature of early ecdysozoans. *Nature* **523**, 75–78 (2015).

38. Daley, A. C. & Bergström, J. The oral cone of *Anomalocaris* is not a classic ‘‘peytoia’’. *Naturwissenschaften* **99,** 501–504 (2012).

39. Daley, A. C., Paterson, J. R., Edgecombe, G. D., García-Bellido, D. C. & Jago, J. B. New anatomical information on *Anomalocaris* from the Cambrian Emu Bay Shale of South Australia and a reassessment of its inferred predatory habits. *Palaeontology* **56,** 971–990 (2013).

40. Daley, A. C. & Budd, G. E. New anomalocaridid appendages from the Burgess Shale, Canada. *Palaeontology* **53**, 721–738 (2010).

41. Whittington, H. B. The lobopod animal *Aysheaia pedunculata* Walcott, Middle Cambrian, Burgess Shale, British Columbia. *Philos. Trans. R. Soc. B Biol. Sci.* **284**, 165–197 (1978).

42. Budd, G. E. The morphology and phylogenetic significance of *Kerygmachela kierkegaardi* Budd (Buen Formation, Lower Cambrian, N Greenland). *Trans. R. Soc. Edinburgh* **89**, 249–290 (1999).

43. Garm, A. & Watling, L. in *The Natural History of the Crustacea, Volume 1, Functional Morphology and Diversity* (eds Watling, L. & Thiel, M.) 167–197 (Oxford University Press, Oxford, 2013).

44. Hou, X.-G., Siveter, D. J., Siveter, D. J., Aldridge, R. J., Cong, P.-Y., Gabbott, S. E., Ma, Z.-Y., Purnell, M. A. & Williams, M. *The Cambrian Fossils of Chengjiang, China – The Flowering of Early Animal Life, Second Edition* (Wiley and Sons, Chichester and Oxford, 2017).

45. Lerosey-Aubril, R., Hegna, T. A., Babcock, L. E., Bonino, E. & Kier, C. Arthropod appendages from the Weeks Formation Konservat-Lagerstätte: new occurrences of anomalocaridids in the Cambrian of Utah, USA. *Bull. Geosci.* **89**, 269–282 (2014).

46. Briggs, D. E. G., Siveter, D. J., Sutton, M. D., Garwood, R. J. & Legg, D. Silurian horseshoe crab illuminates the evolution of arthropod limbs. *Proc. Nat. Acad. Sci.* **109**, 15702–15705 (2012).

47. Aria, C. & Caron, J.-B. Mandibulate convergence in an armoured Cambrian stem chelicerate. *BMC Evol. Biol.* **17**, 261 (2017).

48. Yang, J., Ortega-Hernández, J., Legg, D. A., Lan, T., Hou, J.-b. & Zhang, X.-g. Early Cambrian fuxianhuiids from China reveal origin of the gnathobasic protopodite in euarthropods. *Nat. Comm.* **9**, 470 (2018).

49. Gaines, R. R., Briggs, D. E. G., Orr, P. J. & Van Roy, P. Preservation of giant anomalocaridids in silica-chlorite concretions from the early Ordovician of Morocco. *Palaios* **27**, 317–325 (2012).

50. Van Roy, P. & Briggs, D. E. G. A giant Ordovician anomalocaridid. *Nature* **473**, 510–513 (2011).

51. Yang, J., Ortega-Hernández, J., Butterfield, N. J., & Zhang, X.-g. Specialized appendages in fuxianhuiids and the head organization of early euarthropods. *Nature* **494**, 468–471 (2013).

52. Chen, J.-y., Ramsköld, L. & Zhou, G.-q. Evidence for monophyly and arthropod affinity of Cambrian giant predators. *Science* **264**, 1304–1308 (1994).

53. Hou, X. & Bergström, J. Arthropods of the Lower Cambrian Chengjiang fauna, southwest China. *Fossils Strata* **45**, 1–116 (1997).

54. Jago, J. B., Garcia-Bellído, D. C. & Gehling, J. G. An early Cambrian chelicerate from the Emu Bay Shale, South Australia. *Palaeontology* **59**, 549–562 (2016).

55. Zeng, H., Zhao, F.-C., Yin, Z.-J & Zhu, M.-Y. Appendages of an early Cambrian metadoxidid trilobite from Yunnan, SW China support mandibulate affinities of trilobites and artiopods. *Geol. Mag.* **154**, 1306–1328 (2017).

56. Zacaï, A., Vannier, J. & Lerosey-Aubril, R. Reconstructing the diet of a 505-million-year-old arthropod: *Sidneyia inexpectans* from the Burgess Shale fauna. *Arthropod Struct. Dev.* **45**, 200–220 (2016).
57. Pates, S. & Daley, A. C. *Caryosyntrips*: a radiodontan from the Cambrian of Spain, USA and Canada. *Pap. Palaeontol.* **3**, 461–470 (2017).
58. Whittington, H. B. Trilobites with appendages from the Middle Cambrian Burgess Shale, British Columbia. *Fossils Strata* **4**, 97–136 (1975).
59. Whittington, H. B. Exoskeleton, moult stage, appendage morphology, and habits of the Middle Cambrian trilobite *Olenoides serratus*. *Palaeontology* **23**, 171–204 (1980).
60. Hintze, L. F. *Geologic map of Utah, scale 1:500,000* (Utah Geological and Mineral Survey, Salt Lake City, 1980).
